# Arginase 2 Promotes Colorectal Cancer Metastasis via PI3K/AKT Pathway Activation and Regulates Tumor Immune Infiltration

**DOI:** 10.1002/cam4.71567

**Published:** 2026-02-03

**Authors:** Yueyan Zhang, Ning Qu, Jiale Mei, Caixia Mo, Luojuan Wei, Haixian Shen, Shanfei Zhou, Jinmin Hu, Wenqi Luo, Xianwei Mo

**Affiliations:** ^1^ Colorectal and Anal Disease Unit, Department of Gastrointestinal Surgery Guangxi Medical University Cancer Hospital Nanning Guangxi China; ^2^ Guangxi Key Laboratory of Basic and Translational Research for Colorectal Cancer Nanning Guangxi China; ^3^ Department of Pathology Guangxi Medical University Cancer Hospital Nanning Guangxi China

**Keywords:** Arginase 2, colorectal cancer, epithelial‐mesenchymal transition (EMT), PI3K/AKT signaling, tumor immune microenvironment

## Abstract

**Background:**

Colorectal cancer (CRC) is a leading cause of cancer‐related deaths, with metabolic reprogramming involved in its pathogenesis. Aberrant Arginase 2 (ARG2) expression is linked to malignant progression, but its role in CRC remains unclear.

**Methods:**

ARG2 expression in CRC and adjacent tissues was analyzed. In vitro experiments were performed after ARG2 knockdown. Mechanistic investigations focused on epithelial‐mesenchymal transition (EMT), PI3K/AKT pathway, immune infiltration, and drug sensitivity.

**Results:**

ARG2 was upregulated in CRC tissues, correlating with poor prognosis. ARG2 knockdown inhibited CRC cell proliferation, migration, and invasion by reducing Zinc Finger E‐Box Binding Homeobox 1 (ZEB1), N‐cadherin, and MMP2, suppressing EMT. Additionally, ARG2 knockdown significantly inhibited the PI3K/AKT signaling pathway. Immune infiltration analysis revealed high ARG2 expression correlated with reduced activated B cells and macrophages. Drug sensitivity analysis indicated that high ARG2 expression was associated with decreased efficacy of certain chemotherapeutic agents.

**Conclusion:**

ARG2 is an independent prognostic marker for CRC. It promotes CRC progression via regulating EMT and PI3K/AKT, holding potential as a novel diagnostic and therapeutic target.

**Abbreviations**
ARG2: Arginase 2
AUC: area under curve
BP: biological processes
CC: cellular component
CRC: colorectal cancer
DFS: disease‐free survival
dMMR: deficient mismatch repair
EMT: epithelial‐mesenchymal transition
FDR: false discovery rate
GDSC: Genomics of Drug Sensitivity in Cancer
GEO: Gene Expression Omnibus
GO: Gene Ontology
GSEA: Gene Set Enrichment Analysis
HR: hazard ratio
IC_50_
: half‐maximal inhibitory concentration
ICIs: immune checkpoint inhibitors
IHC: immunohistochemistry
ITM: immunosuppressive tumor microenvironment
KEGG: Kyoto Encyclopedia of Genes and Genomes
MF: molecular function
MMR: mismatch repair
NC: negative control
OS: overall survival
PIP2: phosphatidylinositol‐4,5‐bisphosphate
PIP3: phosphatidylinositol‐3,4,5‐trisphosphate
pMMR: proficient mismatch repair
PTEN: Phosphatase and Tensin Homolog
RNA‐seq: RNA sequencing
ROC: receiver operating characteristic
shRNAs: short hairpin RNAs
ssGSEA: single‐sample gene‐set enrichment analysis
TCGA: The Cancer Genome Atlas
TIICs: tumor‐infiltrating immune cells
TIME: tumor immune microenvironment
ZEB1: Zinc Finger E‐Box Binding Homeobox 1

## 1 Introduction

Global burden analyses position CRC as the second‐leading cause of cancer mortality, with over 1.9 million new diagnoses and approximately 904,000 deaths recorded in 2022 alone [1]. Despite significant advancements in diagnostic and therapeutic approaches, cure rates and long‐term survival in CRC patients remain suboptimal, particularly for those with metastatic CRC [2, 3]. Tumor heterogeneity, an immunosuppressive tumor microenvironment (ITM), and the emergence of drug resistance collectively exacerbate the therapeutic challenges in CRC [4, 5, 6, 7, 8]. It is predicted that the number of CRC cases will surge to 4.7 million by 2070 [9], underscoring the urgency to identify novel therapeutic targets.

ARG2, a mitochondrial enzyme widely expressed in extrahepatic tissues (predominantly in the kidney, prostate, digestive and gastrointestinal tracts, muscle, and endocrine tissues), is encoded by a gene located on human chromosome 14q24 [10]. The mitochondrial enzyme ARG2 catabolizes l‐arginine into l‐ornithine and urea [11], creating an immunosuppressive milieu via extracellular arginine depletion—a mechanism exploited by tumors [12]. Notably, dysregulation of ARG2 has been observed in various malignant tumors, suggesting its potential role in tumorigenesis. For instance, in glioblastoma, downregulation of ARG2 via MicroRNA‐613 has been shown to reduce tumor cell growth, colony formation, and metastatic potential [13]. Thyroid cancer exhibited elevated ARG2 levels, and silencing ARG2 enhanced cancer cell apoptosis while reducing proliferation [14]. Within cancer‐associated fibroblasts, ARG2 expression correlated with tissue hypoxia in pancreatic cancer patients and served as a predictor of poor prognosis [15]. Melanoma showed upregulated ARG2, which diminished sorafenib‐induced cell death [16]. Thus, ARG2 exhibited multifaceted roles in cancer, with its influence on tumor proliferation, migration, apoptosis, drug resistance, and the tumor immune microenvironment (TIME) increasingly recognized. While Leu and Wang [17] noted ARG2 elevation in CRC [17], its mechanistic role in metastasis and immune modulation remains unexplored, prompting this investigation.

In this study, we conducted a comprehensive analysis of ARG2 expression in CRC tissues and further investigated its associations with clinicopathological characteristics and patient prognosis. Through enrichment analysis and functional assays, we examined the effects of ARG2 on CRC cell proliferation, migration, and invasion, elucidating the underlying mechanisms. This study could provide novel mechanistic insights into the role of ARG2 in CRC initiation and progression, offering valuable guidance for optimizing therapeutic strategies and improving prognostic outcomes in CRC.

## 2 Material and Methods

### 2.1 Data Collection and Processing

Gene expression matrices for CRC patients were retrieved from The Cancer Genome Atlas (TCGA) database (https://www.cancer.gov/ccg/research/genome‐sequencing/tcga), comprising 41 normal and 473 CRC samples. Additionally, datasets GSE113513 (14 normal and 14 CRC samples), GSE41015 (12 normal and 19 CRC samples), and GSE24551‐GPL5175 (13 normal and 160 CRC samples) were obtained from the Gene Expression Omnibus (GEO) database (https://www.ncbi.nlm.nih.gov/geo/). All RNA sequencing (RNA‐seq) data requiring normalization were log2‐transformed prior to analysis.

### 2.2 Clinical Samples Collection

We collected 171 CRC tissues and 75 adjacent normal tissues from CRC patients at the Colorectal and Anal Disease Unit, Department of Gastrointestinal Surgery, Guangxi Medical University Cancer Hospital through surgical removal. None of the patients included in this study had received any radiotherapy, chemotherapy, or other antitumor treatments prior to tissue collection. All tissues were immediately snap‐frozen in liquid nitrogen post‐surgery and stored at −80°C. Comprehensive clinicopathological data, including sex, age, tumor size, cancer embolus, nerve invasion, T stage, N stage, M stage, Stage, mismatch repair (MMR) status, and tumor budding, were recorded. Survival information was obtained through follow‐up. Among the 171 enrolled patients, 28 were lost to follow‐up, resulting in 143 patients with complete clinical information for subsequent analysis. Ethical approval for this study was granted by the Ethics Committee of the Guangxi Medical University Cancer Hospital. Written informed consent for the use of their specimens and information was obtained from all patients.

### 2.3 Immunohistochemistry (IHC) Staining

Three‐micrometer‐thick sections were prepared from paraffin‐embedded tissues of 171 CRC patients and 75 adjacent normal tissues. After deparaffinization with graded ethanol and rehydration, sections were blocked at room temperature and incubated with anti‐ARG2 primary antibody (1:200; A19233, ABclonal) at 4°C overnight. After washing with PBS, sections were incubated with HRP‐conjugated secondary antibody (RK50015, ABclonal) for 1 h at room temperature, followed by staining with DAB and hematoxylin. According to the IHC scoring criteria, 10 different fields per sample were evaluated for IHC staining by two independent pathologists (WQ. L and RW. M). The scoring system was as follows: staining intensity was scored from 0 to 3 (negative, weak, moderate, and strong, respectively). IHC scoring integrated intensity (0–3) and positivity extent (0–4 scales), with final scores representing the mean of 10 fields assessed independently by two pathologists. The score for each field was calculated as the product of the intensity and proportion scores, and the final score for each sample was determined as the arithmetic mean of the scores from the 10 fields.

### 2.4 Survival Analysis

Survival data from the GSE106584 dataset were retrieved using the “GEOquery” R package. CRC patients in this dataset were categorized into low‐ and high‐ARG2 expression groups based on the median ARG2 mRNA expression level, and Kaplan–Meier analysis with log‐rank tests was performed to assess the impact of ARG2 mRNA levels on survival outcomes. To further analyze the prognostic value of ARG2 protein, we divided 143 CRC patients into two groups based on IHC scores for survival analysis. An IHC score < 6 was considered low expression; otherwise, it was considered high expression. In addition, we performed survival analyses of CRC patients with different clinicopathological characteristics.

### 2.5 Nomogram Construction and Analysis

We performed univariate and multivariate Cox regression analyses of ARG2 protein expression and clinicopathological characteristics of CRC patients using the “survival” R package, and visualized the results using the “forestplot” R package. Based on multivariate Cox regression outcomes, a nomogram model for predicting CRC prognosis was constructed using the “rms” R package. Model performance was evaluated through calibration curves and receiver operating characteristic (ROC) curves.

### 2.6 Cell Culture and Transfection

Human CRC cell lines (DLD1, LoVo, HCT116, and SW480) and the normal colon epithelial cell line (NCM460) were purchased from the ATCC. All cell lines were maintained in DMEM medium (Gibco) containing 10% fetal bovine serum and 1% penicillin–streptomycin, under 37°C and 5% CO_2_ humidified conditions. Cells in the logarithmic growth phase were used for follow‐up experiments. All cell lines were authenticated by STR analysis and validated to be free of mycoplasma contamination before usage in this study. Two short hairpin RNAs (shRNAs) targeting ARG2 were designed and synthesized for knockdown studies, with a scrambled shRNA serving as the negative control (NC). Cells were transduced with lentiviral vectors according to the manufacturer's instructions. The efficiency of ARG2 knockdown was assessed by Western blot analysis.

### 2.7 Western Blot

Cell samples were lysed using NP‐40 lysis buffer (P0013F, Beyotime) supplemented with protease inhibitors (HYK0010, MCE) and phosphatase inhibitors I (HY‐K0021, MCE) and II (HY‐K0022, MCE) to extract total protein. For tissue samples, frozen CRC or adjacent normal tissue was homogenized in the same NP‐40 lysis buffer supplemented with protease inhibitors and phosphatase inhibitors I/II using a freezing grinder. The homogenate was centrifuged at 13,000 rpm for 15 min at 4°C, and the supernatant was collected as total protein. Protein concentration was quantified using a BCA assay kit. Equal amounts of protein (50 μg per lane) were resolved by 10% SDS‐PAGE and electrophoretically transferred to PVDF membranes. Membranes were blocked with 5% (w/v) non‐fat milk in TBST for 1 h at room temperature, followed by overnight incubation at 4°C with primary antibodies diluted in blocking buffer/TBST: ARG2 (1:200; A19233, ABclonal), ZEB1 (1:1000; A5600, ABclonal), N‐cadherin (1:1000; A19083, ABclonal), MMP2 (1:1000; A19080, ABclonal), AKT (1:1000; A17909, ABclonal), p‐AKT (1:1000; AP1208, ABclonal), p‐PI3K (1:1000; AP0854, ABclonal), mTOR (1:1000; A2445, ABclonal), p‐mTOR (1:1000; AP0115, ABclonal), and GAPDH (1:10000; A19056, ABclonal). After washing with TBST, membranes were incubated for 2 h at room temperature with HRP‐conjugated Goat anti‐Rabbit IgG (H + L) secondary antibody (AS014, ABclonal) diluted 1:5000 in blocking buffer/TBST. Following extensive TBST washes, protein bands were visualized using an ECL chemiluminescence reagent kit (BMU102, Abbkine) according to the manufacturer's instructions.

### 2.8 CCK‐8 Assay

Cell viability was assessed using the CCK‐8 method. 5 × 10^3^ cells were seeded in 96‐well plates and cell viability was assessed at Days 0, 1, 2, 3, and 4 post‐treatment. At each time point, 10 μL of CCK‐8 solution was added to each well and incubated for 2 h. The absorbance was measured at 450 nm using a microplate reader to evaluate cell viability. Since the absorbance value at 450 nm is positively correlated with the number of viable cells, the “relative cell number” (used in Figure 4D,E) was calculated as the ratio of the absorbance value at each time point (Days 1–4) to that at Day 0 (Day 0 absorbance = 1). This normalization allows for intuitive visualization of cell proliferation trends.

### 2.9 Colony Formation Assay

CRC cells were seeded at a density of 800 cells per well in six‐well plates and cells were cultured at 37°C in a humidified atmosphere with 5% CO_2_ for 14 days to allow colony formation. After incubation, colonies were washed twice with PBS, fixed with 4% paraformaldehyde (G1101, Servicebio) for 20 min, followed by staining with 0.1% crystal violet for 30 min at room temperature. Counting of colonies with more than 50 cells utilized GraphPad Prism 8.0.

### 2.10 Wound‐Healing Assay

CRC cells were plated at a density of 1 × 10^6^ cells per well in six‐well plates and incubated overnight. Subsequently, a sterile 10 μL pipette tip was used to create a linear scratch. Cell migration toward the scratch area was monitored by capturing images of the same wound region using a microscope at 0 and 24 h.

### 2.11 Transwell Assay

The invasive ability of CRC cells was evaluated using a Matrigel‐coated transwell chamber (Costar). A total of 1 × 10^5^ cells were suspended in 200 μL of FBS‐free medium and seeded into the upper chamber of each well, while 700 μL of medium containing 10% FBS was added to the lower chamber. After 24 h of culture, the non‐invasive cells were removed, fixed with methyl alcohol, stained with 0.1% crystal violet for 30 min, and photographed under an optical microscope.

### 2.12 Enrichment Analysis

To identify genes co‐expressed with ARG2, we used the LinkedOmics platform (https://www.linkedomics.org/login.php) to perform Spearman tests on the CRC cohort. When the | *r* | ≥ 0.25 and *p* < 0.05, the correlation was considered statistically significant. We used the “clusterProfiler” R package to conduct the Gene Ontology (GO) and Kyoto Encyclopedia of Genes and Genomes (KEGG) analysis for the co‐expressed genes of ARG2. In addition, we obtained the hallmark gene sets (hall v2024.1.Hs.symbols pathways) from the Gene Set Enrichment Analysis (GSEA) website (https://www.gsea‐msigdb.org/gsea/msigdb/index.jsp). GSEA was performed for the co‐expressed genes of ARG2. When the *p* < 0.05 and the false discovery rate (FDR) < 0.25, it was statistically significant.

### 2.13 Immune Infiltration Analysis

We used the “GSVA” R package for single‐sample gene‐set enrichment analysis (ssGSEA) to obtain ssGSEA scores for 28 tumor‐infiltrating immune cells (TIICs). Comparison of differences between ARG2 low‐ and high‐expression groups in TCGA database followed. Exploration of correlations between ARG2 expression and 28 immune cell types utilized the “linkET” R package.

### 2.14 Drug Sensitivity Analysis

We predicted chemotherapy responses in CRC samples from TCGA database based on the Genomics of Drug Sensitivity in Cancer (GDSC) database. Specifically, the half‐maximal inhibitory concentration (IC_50_) values for various chemotherapeutic agents were computationally predicted using the “oncoPredict” R package, which maps the gene expression profiles of our tumor samples onto GDSC's pre‐trained drug sensitivity models. By comparing the IC_50_ values between the low‐ and high‐expression groups of ARG2, potential drugs whose sensitivity was affected by ARG2 were screened.

### 2.15 Statistical Analysis

Analysis of differences between two groups utilized the Wilcoxon rank‐sum test, with paired *t*‐tests (two‐sided) for paired samples. Evaluation of associations between ARG2 expression and clinicopathological characteristics employed chi‐square tests. Estimation of overall survival (OS) and disease‐free survival (DFS) used Kaplan–Meier analysis with log‐rank tests. Cox regression analysis was used to calculate hazard ratios (HRs). Assessment of variable correlations used Pearson or Spearman tests based on data distribution. All experiments in this study were independently repeated at least twice, and the data were represented as mean ± SD. Statistical significance was calculated using R 4.4.2, GraphPad Prism 8.0, and SPSS 17.0. *p* < 0.05 was considered statistically significant. Statistical significance was defined as *p < 0.05, **p < 0.01, ***p < 0.001, ****p < 0.0001, or no significance (ns). All statistical details of the experiments can be found in the figure legends.

## 3 Results

### 3.1 Expression and Diagnostic Value of ARG2

**FIGURE 1:**
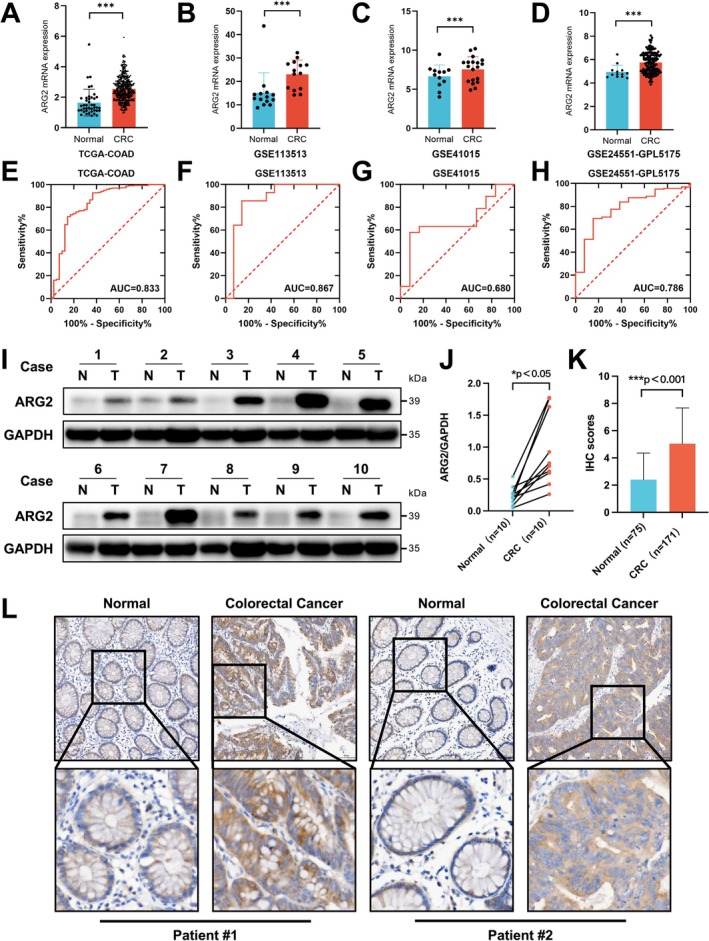
ARG2 expression is up‐regulated in CRC. (A–D) The expression of ARG2 mRNA in CRC was analyzed in publicly available databases, including the TCGA (TCGA‐COAD) and GEO microarray datasets (GSE113513, GSE41015, and GSE24551). (E–H) ROC curves of ARG2 expression for the differentiation of CRC from para‐cancerous tissues based on corresponding datasets. (I, J) The protein level of ARG2 expression was assessed by Western blot in 10 pairs of randomly selected CRC and para‐cancerous tissues. (K, L) ARG2 protein expression was assessed by IHC staining in 171 CRC patients. Typical pictures are shown (IHC, ×200). Data are presented as mean ± SD. Statistical significance was determined by Wilcoxon rank‐sum test (A–D, K) and paired *t*‐tests (two‐sided) (J). *p < 0.05, ***p < 0.001.

mRNA expression analysis of ARG2 in CRC and normal tissues using TCGA and GEO datasets (GSE113513, GSE41015, GSE24551) showed consistent upregulation in CRC tissues, with statistical significance (Figure 1A–D). ROC curve analysis showed diagnostic performance for ARG2 mRNA expression in CRC, with area under curve (AUC) values of 0.833, 0.867, 0.680, and 0.786 (Figure 1E–H). Western blot analysis confirmed elevated ARG2 protein levels in CRC tissues compared to adjacent normal tissues (Figure 1I,J). IHC staining showed that the ARG2 staining intensity and the proportion of positive cells in CRC tissues were significantly higher than those in adjacent normal tissues, further confirming upregulated ARG2 protein expression. The positive signals were mainly concentrated in the cell membrane and cytoplasm (Figure 1K,L).

### 3.2 Prognostic Value of ARG2

**FIGURE 2:**
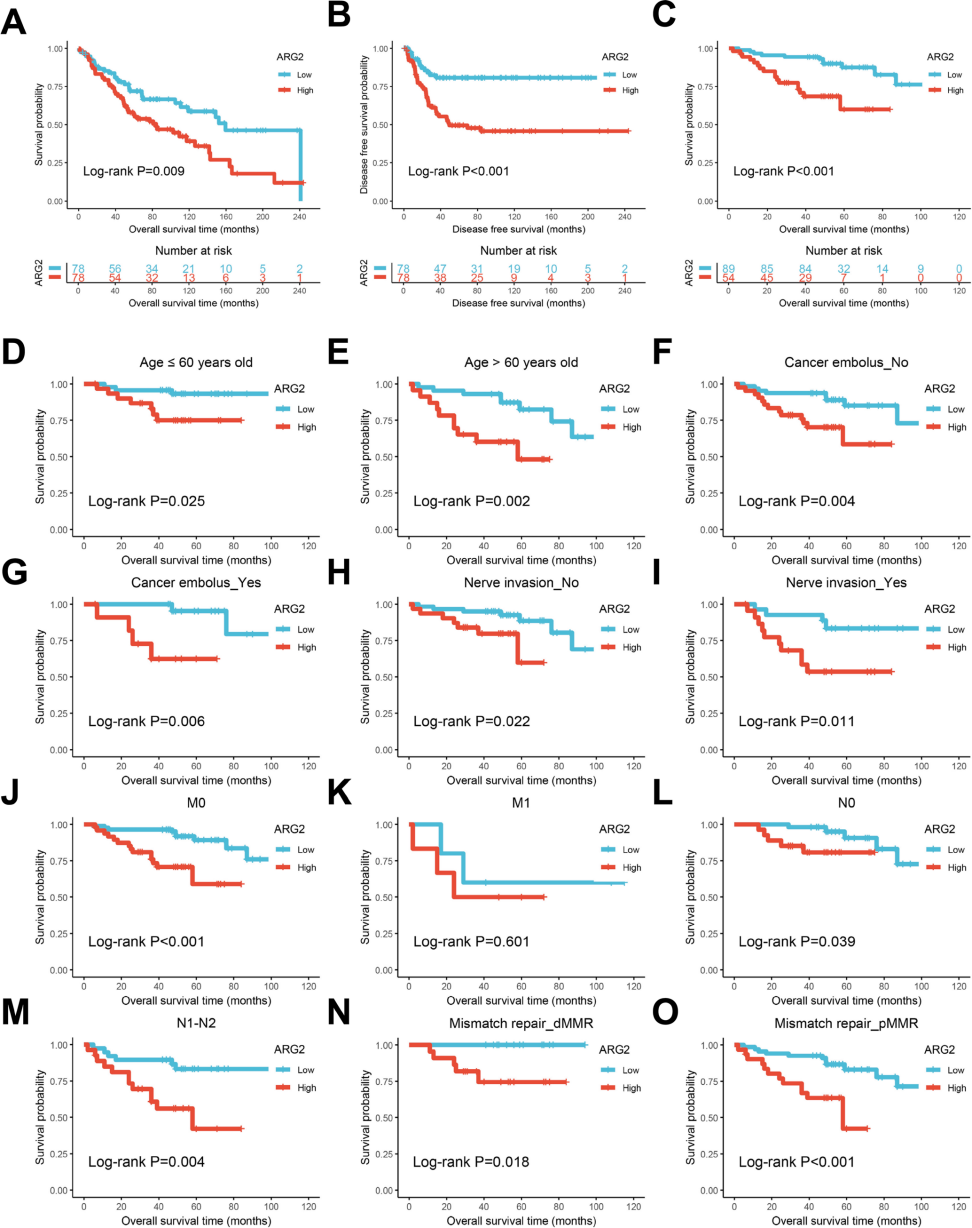
High expression of ARG2 in CRC was associated with poor prognosis. Survival curves of overall survival (OS) (A) and disease free survival (DFS) (B) in GSE106584. (C) Kaplan–Meier survival curves of ARG2 in our cohort. Kaplan–Meier survival curves for subgroups of ARG2 high and ARG2 low groups in ≤ 60 (D) and > 60 years old (E), cancer embolus: no (F) and yes (G), nerve invasion: no (H) and yes (I), M0 (J) and M1 (K), N0 (L) and N1‐2 (M), and Mismatch repair: dMMR (N) and pMMR (O). Data are presented as Kaplan–Meier survival curves. All survival analyses were performed using the log‐rank test. The log‐rank *p* value indicated in each panel.

Table S1 summarizes the clinicopathological characteristics of the CRC cohort. Chi‐square tests showed a significantly higher proportion of deficient mismatch repair (dMMR) in the high ARG2 expression group compared to the low expression group. Kaplan–Meier analysis of the GSE106584 dataset revealed that elevated ARG2 mRNA expression was associated with reduced OS and DFS in CRC patients (Figure 2A,B). In 143 CRC patients, high ARG2 protein expression significantly correlated with poorer OS (Figure 2C). Subgroup analyses demonstrated the prognostic value of ARG2 across clinicopathological features, including age (≤ 60 and > 60 years old), cancer embolus (no and yes), nerve invasion (no and yes), N0 and N1‐N2 stages, M0 stages, and MMR status, consistently indicating worse outcomes in the high‐expression group (Figure 2D–O).

### 3.3 Nomogram Model for Prognostic Prediction in CRC

**FIGURE 3:**
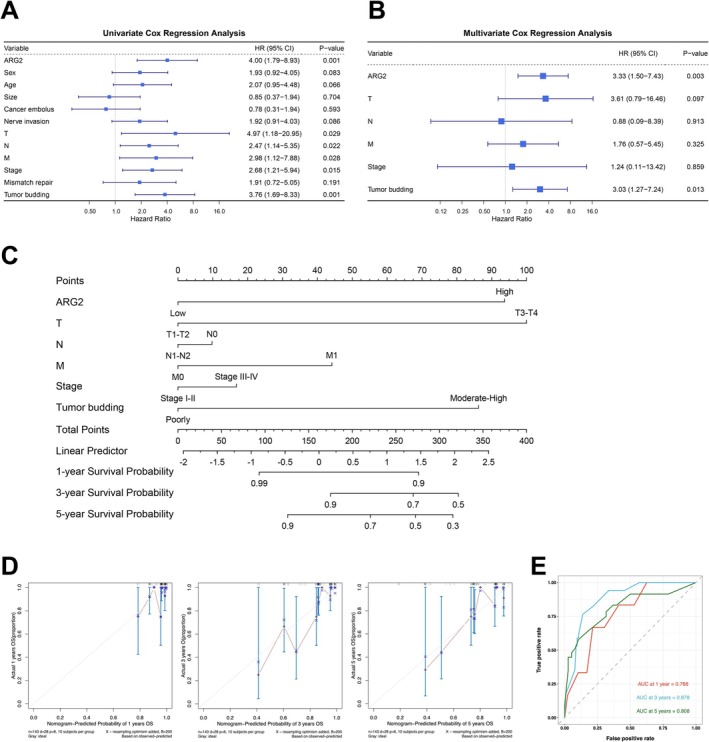
Prognostic impact of ARG2 in CRC patient cohort. Forest map based on univariate Cox regression (A) and multivariate Cox analysis (B) for overall survival (OS). (C) Nomogram based on clinical characteristics and ARG2 expression. (D) A calibration curves for prediction of 1‐, 3‐, 5‐year OS rates of patients with CRC. (E) Time‐dependent ROC curve for risk score models in the cohort. Data are presented as forest plots, nomogram, calibration plots, and ROC curves. Statistical significance was determined by Cox regression analysis, with *p* < 0.05 considered significant.

Univariate Cox regression identified ARG2 expression, T, N, M stages, Stage, and tumor budding as CRC risk factors (Figure 3A). Multivariate Cox regression confirmed ARG2 as an independent prognostic biomarker for OS (Figure 3B). A nomogram model was developed based on multivariate Cox regression results to quantify risk and predict survival probability. ARG2 expression exhibited a strong influence on survival prediction, accurately forecasting 1‐, 3‐, and 5‐year outcomes (Figure 3C). Calibration curves validated the predictive performance of the model, and AUC values of 0.768, 0.878, and 0.808 for 1‐, 3‐, and 5‐year predictions indicated good predictive performance across multiple time points (Figure 3D,E).

### 3.4 Knockdown of ARG2 Suppresses CRC Cell Proliferation

**FIGURE 4:**
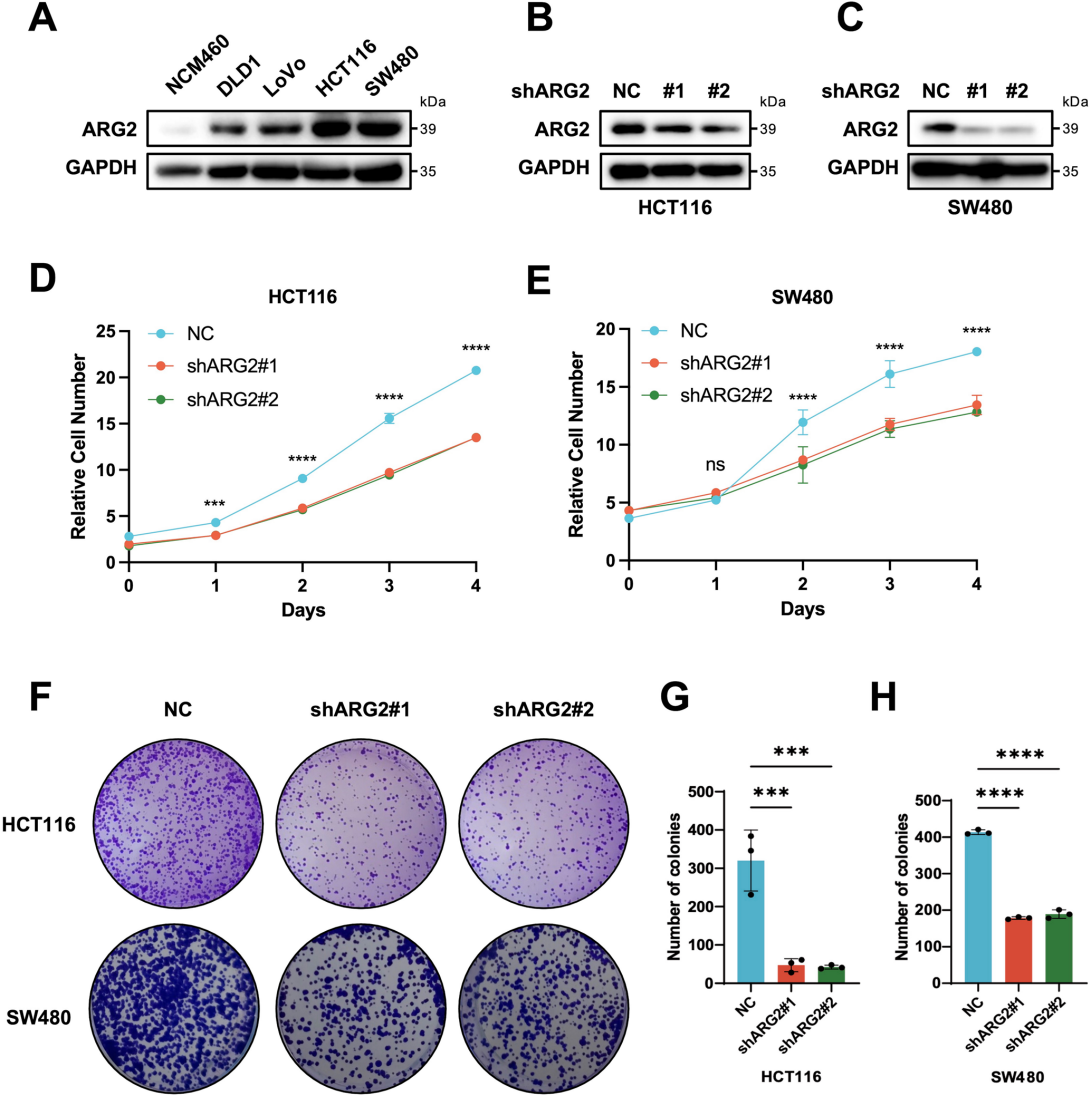
Knockdown of ARG2 inhibits the proliferative ability of CRC cells. (A) ARG2 protein expression in CRC cell lines and normal intestinal epithelial cells was determined by Western blot. Western blot was also used to detect the knockdown efficiency of ARG2 protein in HCT116 (B) and SW480 (C) cells. (D, E) CCK‐8 assay analysis of proliferation in ARG2‐knockdown (shARG2) and NC HCT116/SW480 cells. “Relative cell number” in (D) and (E) is derived from CCK‐8 assay results, representing the ratio of absorbance at each time point to Day 0 (baseline). It reflects the relative change in viable cell quantity over the culture period. (F–H) Colony formation assay to evaluate proliferation of shARG2 and NC HCT116/SW480 cells. Data in (D, E) and (F–H) are presented as mean ± SD of three independent experiments. Statistical significance was determined by two‐way ANOVA (D, E) and one‐way ANOVA (G, H). ns, no significant difference. ***p < 0.001, ****p < 0.0001.

HCT116 and SW480 cells, which exhibit relatively high ARG2 expression, were selected for lentiviral transfection (Figure 4A). Western blot data verified successful ARG2 knockdown in HCT116 and SW480 cells (Figure 4B,C). CCK‐8 assays showed that ARG2 knockdown significantly reduced proliferation in both cells. After 1 day of culture for HCT116 cells and 2 days for SW480 cells, the cell growth fold change in the ARG2 knockdown groups was significantly lower than that in the NC group (Figure 4D,E). Colony formation assays further confirmed that ARG2 knockdown inhibited CRC cell proliferation, with fewer colonies in the knockdown groups for both cells compared to the NC group (Figure 4F–H).

### 3.5 Knockdown of ARG2 Inhibits CRC Cell Migration and Invasion

**FIGURE 5:**
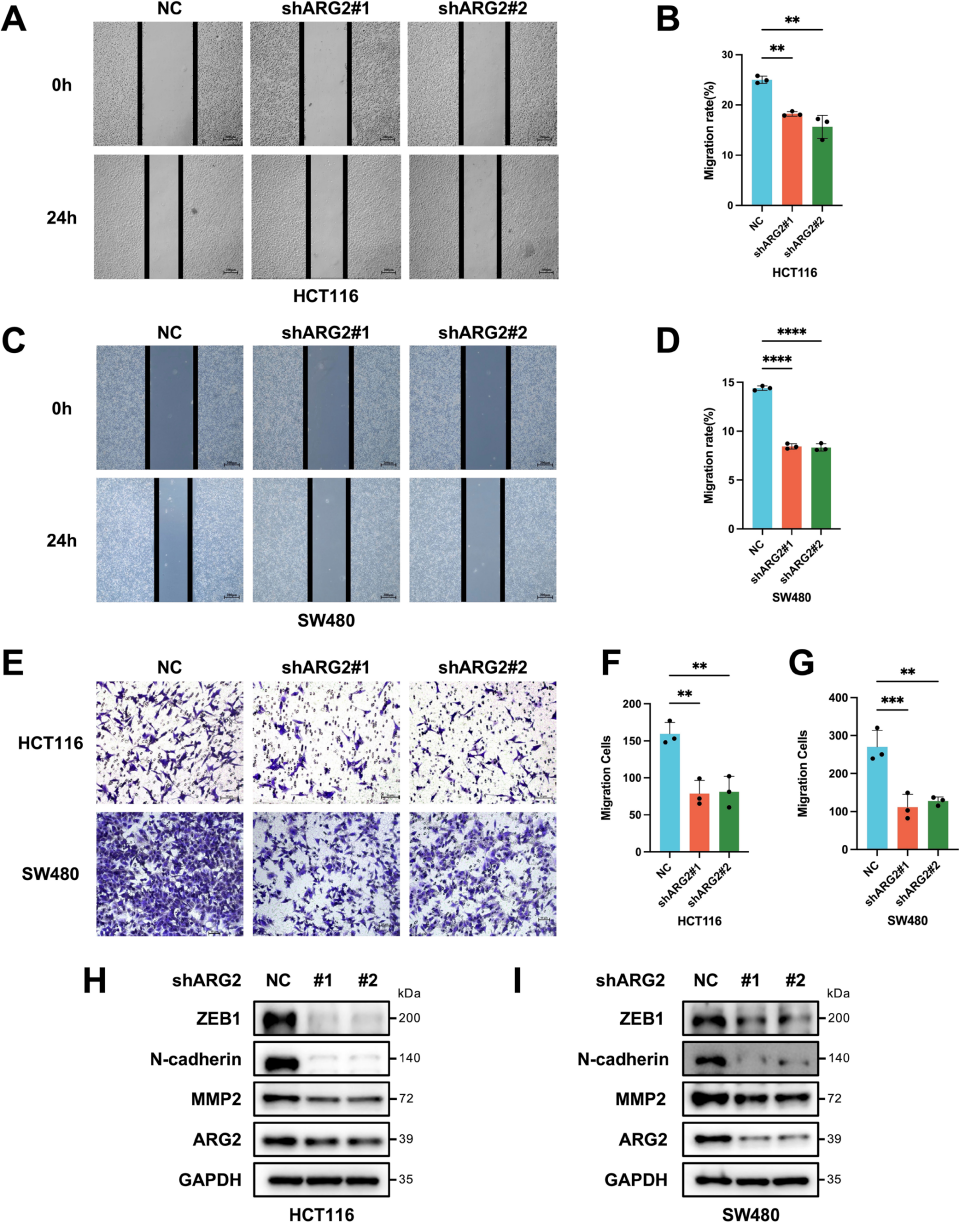
Knockdown of ARG2 suppresses invasion and migration capabilities of CRC cells in vitro and restrains EMT progression. (A–D) Wound‐healing assay was performed to evaluate the migration capacity of shARG2 and NC HCT116/SW480 cells. The wound closure after 24 h post scratching was measured. Scale bar represents 200 μm. (E–G) Transwell assay was used to assess the invasion capacity of shARG2 and NC HCT116/SW480 cells. Scale bar represents 50 μm. (H, I) The protein expressions of EMT markers in shARG2 and NC HCT116/SW480 cells, including ZEB1, N‐cadherin, and MMP2, were measured by western blotting. Data in (A–G) are presented as mean ± SD of three independent experiments. Statistical significance was determined by one‐way ANOVA (B, D, F, G). ns, no significant difference. **p < 0.01, ***p < 0.001, ****p < 0.0001.

Wound‐healing and Transwell assays demonstrated that ARG2 knockdown markedly reduced migration and invasion capacities in HCT116 and SW480 cells (Figure 5A–G). As CRC invasion and migration are associated with EMT [18, 19], the impact of ARG2 on EMT‐related proteins, including ZEB1, N‐cadherin, and MMP2 was examined. Western blot analysis showed that ARG2 knockdown in HCT116 and SW480 cells significantly reduced ZEB1, N‐cadherin, and MMP2 compared to the NC group (Figure 5H,I). These results suggested that ARG2 knockdown inhibited CRC cell migration and invasion by modulating the EMT pathway.

### 3.6 ARG2 Promotes CRC Progression via the PI3K/AKT Signaling Pathway

**FIGURE 6:**
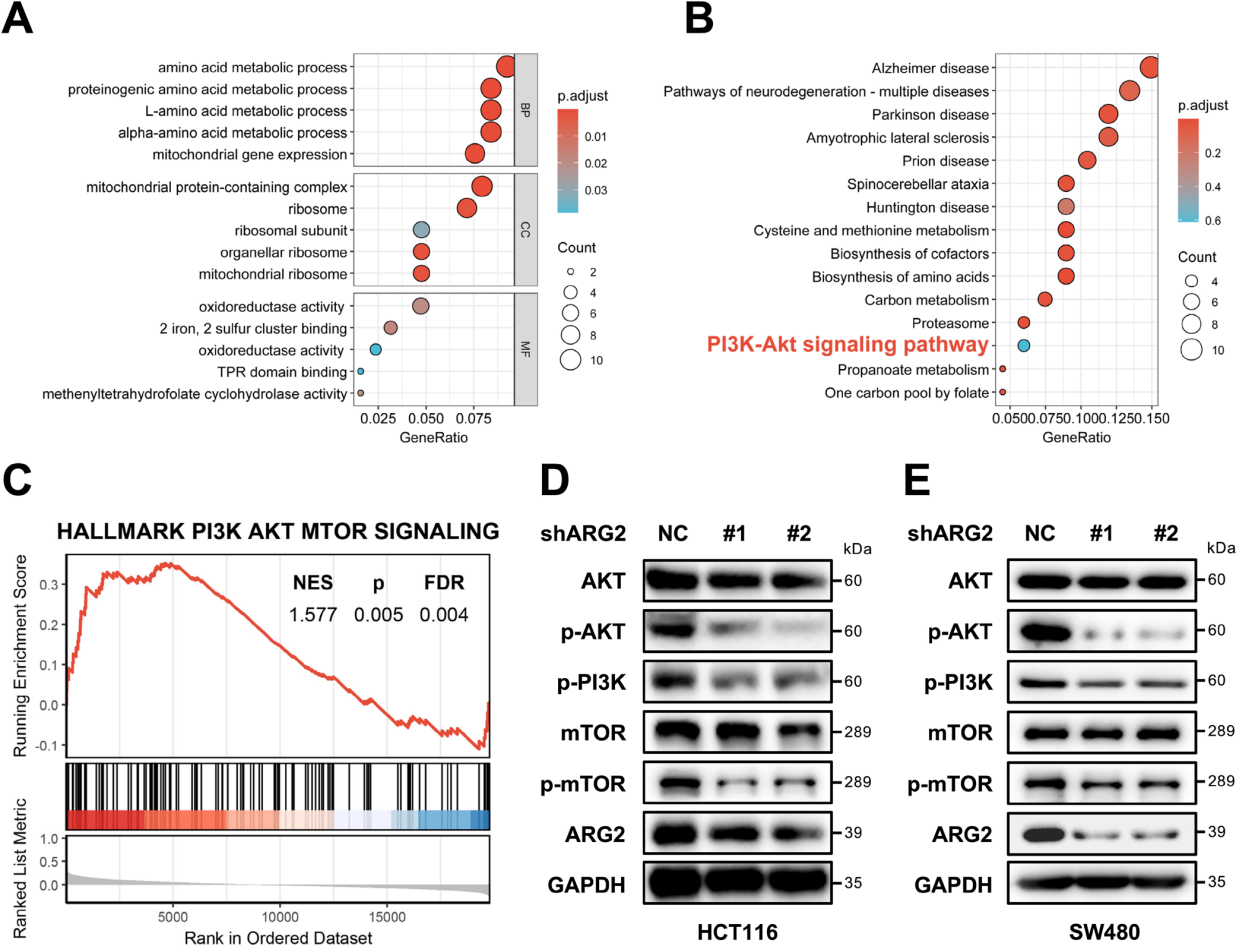
GO and KEGG enrichment analyses revealed that ARG2 is involved in the regulation of the PI3K/AKT signaling pathway in CRC. (A) The GO bubble plot illustrates the top enriched Gene Ontology terms across biological processes (BP), cellular components (CC), and molecular functions (MF). (B) The KEGG analysis highlights significantly enriched pathways, among which the PI3K/AKT signaling pathway was involved. (C) GSEA analysis revealed a significant association between ARG2 expression and the PI3K/AKT signaling pathway. (D, E) Protein levels of total AKT, p‐AKT, p‐PI3K, mTOR, and p‐mTOR were measured by western blotting in shARG2 and NC HCT116/SW480 cells. Statistical significance for GSEA was determined by the permutation test, with FDR < 0.05 considered significant.

To investigate the molecular mechanisms of ARG2 in CRC, GO, and KEGG enrichment analyses were performed on genes co‐expressed with ARG2. GO analysis results are presented in Figure 6A, while KEGG analysis revealed significant enrichment in the PI3K/AKT signaling pathway (Figure 6B). GSEA further corroborated this finding, demonstrating enrichment of the HALLMARK PI3K‐AKT‐mTOR signaling pathway in genes positively correlated with ARG2 expression (Figure 6C). Given the established role of the PI3K/AKT pathway in CRC pathogenesis [20], we employed Western blot to verify the role of ARG2 in regulating the PI3K/AKT signaling pathway. And Western blot analysis confirmed that ARG2 knockdown significantly reduced expression of AKT, p‐AKT1, p‐PI3K, mTOR, and p‐mTOR in both HCT116 and SW480 cells compared to the NC group, indicating suppression of the PI3K/AKT pathway (Figure 6D,E). These findings suggested that ARG2 may promote CRC progression by regulating the PI3K/AKT signaling pathway.

### 3.7 Immune Correlations and Chemotherapy Impact of ARG2

**FIGURE 7:**
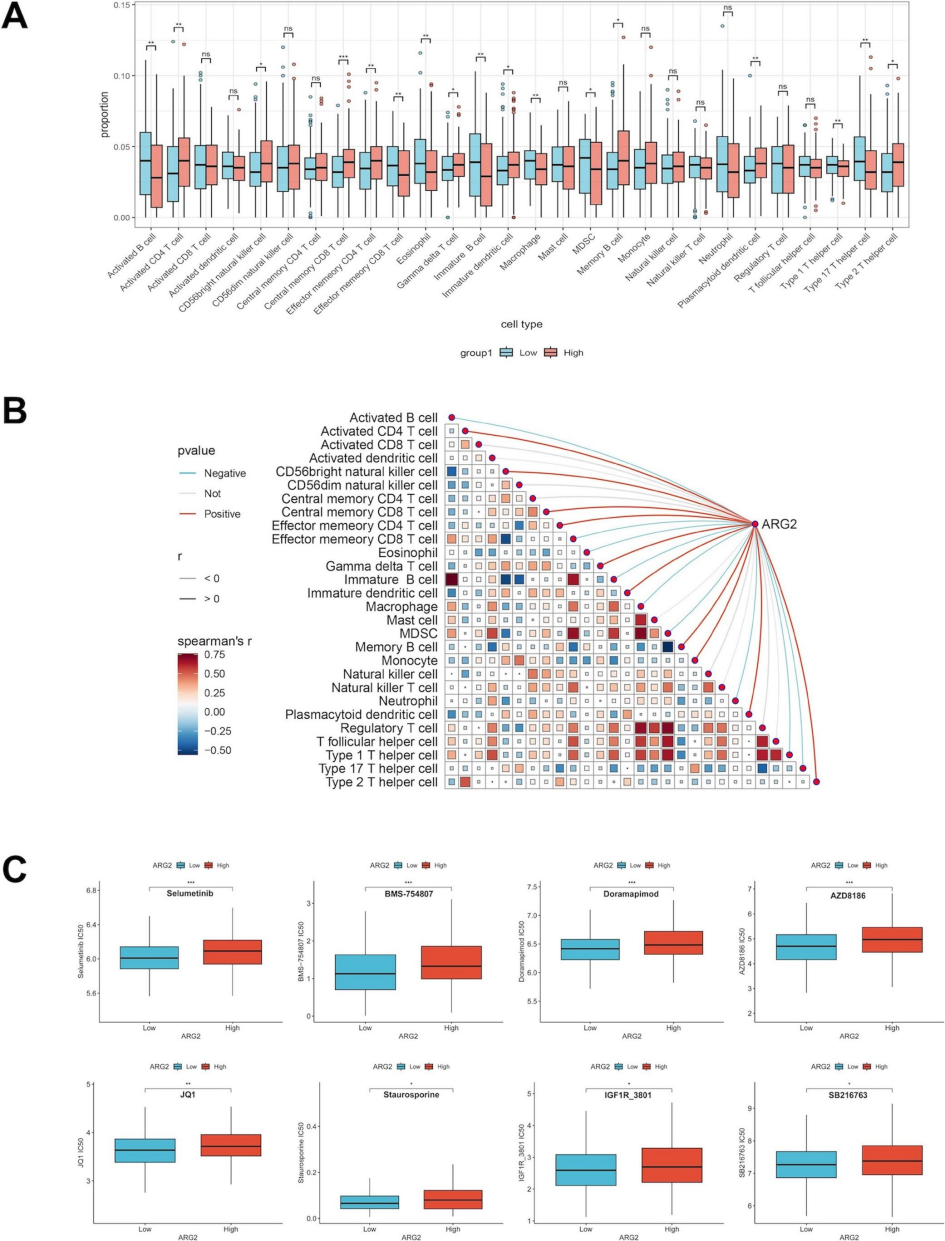
Correlation analysis of ARG2 expression with the TIME and drug sensitivity in CRC. (A) The varied proportions of 28 TIICs abundance by ssGSEA methods between high and low ARG2 expression groups in TCGA database. (B) Correlation between ARG2 expression and common immune cells by “linkET” R package. (C) The predicted IC_50_ values of anti‐CRC therapies drugs according to high and low ARG2 expression. Data are presented as box plots. Statistical significance was determined by the Wilcoxon rank‐sum test. ns, no significant difference. *p < 0.05, **p < 0.01, ***p < 0.001.

Our study showed that CRC patients with high ARG2 expression had a significantly higher proportion of dMMR. And previous studies have demonstrated that dMMR is associated with an enhanced response to immune checkpoint inhibitors (ICIs) [21]. Given the established immunosuppressive role of ARG2 [12], its impact on the TIME of CRC was investigated. The high ARG2 expression group showed significantly lower infiltration of activated B cells and macrophages compared to the low expression group (Figure 7A). Pearson test identified significant associations between ARG2 and 18 immune cell types, with negative correlations observed for activated B cells and macrophages (Figure 7B). Considering the important role of ARG2 in CRC, its influence on chemotherapeutic drug sensitivity was assessed using the GDSC database. Eight drugs (Selumetinib, BMS‐754807, Doramapimod, AZD8186, JQ1, Staurosporine, IGF1R_3801, and SB216763) showed significantly higher IC_50_ values in the high ARG2 expression group compared to the low expression group (Figure 7C), indicating that elevated ARG2 expression was associated with reduced sensitivity to these CRC chemotherapeutic agents.

## 4 Discussion

Maintaining proliferative signals, evading growth inhibitors, resisting cell death, achieving replicative immortality, inducing angiogenesis, activating invasion and metastasis, reprogramming energy metabolism, and evading immune destruction are considered the main hallmarks of cancer [22]. ARG2 has been shown to be involved in proliferation, senescence, apoptosis, autophagy, immunosuppression, and inflammatory responses in various cell types [12, 23, 24, 25, 26]. As a key enzyme in the urea cycle, ARG2 promotes the formation of l‐ornithine. l‐ornithine is further metabolized to produce polyamines essential for cell proliferation, growth, tissue repair, and other physiological functions [27]. Notably, increased polyamine synthesis has been observed in multiple malignancies, including CRC [28, 29, 30]. Additionally, ARG2 competes with nitric oxide synthase for the common substrate l‐arginine, thereby reducing the production of nitric oxide in cells. This contributes to tumor growth and progression [31, 32]. Dysregulated ARG2 expression has been reported in various cancers, including CRC, suggesting its potential role in tumorigenesis, particularly in CRC. However, the precise contributions and underlying mechanisms of ARG2 in CRC remain unclear, prompting our investigation.

Our study confirmed that ARG2 was significantly upregulated at both mRNA and protein levels in CRC tissues compared to normal tissues. This finding aligns with prior reports of ARG2 upregulation in CRC [17] and other malignancies [14, 16, 33], suggesting a general promoting role in tumorigenesis for ARG2. Through analysis of the relationship between ARG2 expression and clinicopathological characteristics, we determined that elevated ARG2 expression significantly correlated with dMMR in CRC patients. Previous studies have shown that dMMR, which leads to microsatellite instability, accounts for approximately 15% of CRC cases [34] and is associated with improved responses to ICIs [21]. Thus, CRC patients with upregulated ARG2 may benefit from ICIs therapy. However, survival analyses revealed that high ARG2 expression was associated with shorter OS and higher recurrence rates, positioning it as a risk factor for prognosis. This discrepancy may be attributed to the low prevalence of dMMR in CRC and the limited number of ICIs‐treated patients in our survival analysis cohort.

Our current study showed that ARG2 knockdown significantly suppressed CRC cell proliferation, migration, and invasion. Moreover, ARG2 downregulation reduced the activation of EMT signaling in CRC cells, indicating that ARG2 may drive CRC progression and metastasis by modulating EMT pathways. In fact, EMT is a dynamic biological process where epithelial cells undergo a reversible shift to a mesenchymal state, which is associated with reduced cell adhesion, loss of polarity, and enhanced migratory and invasive abilities [35]. Initially described in embryonic development, EMT is now recognized as a critical mechanism in cancer progression, particularly in invasion and metastasis [36]. Molecularly, EMT is marked by downregulation of epithelial markers and upregulation of mesenchymal markers, reflecting a shift in cellular identity. E‐cadherin, a key epithelial marker, is essential for maintaining adherens junctions and epithelial integrity [37] but is significantly repressed during EMT via transcriptional suppression mediated by EMT transcription factors such as ZEB1, Snail, Slug, and Twist [38, 39]. Concurrently, mesenchymal markers like Vimentin and N‐cadherin are upregulated, correlating with enhanced invasion and migration [40]. Additionally, MMPs such as MMP2 degrade extracellular matrix components, thereby facilitating tumor invasion and metastasis [36].

Through enrichment and Western blot analyses, we established that ARG2 promotes CRC development by activating the PI3K/AKT signaling pathway. Activation of the PI3K/AKT pathway begins with class I PI3K‐mediated conversion of phosphatidylinositol‐4,5‐bisphosphate (PIP2) to phosphatidylinositol‐3,4,5‐trisphosphate (PIP3), a process that triggers AKT kinase activation via phosphorylation [41]. These events include phosphorylation of key regulators such as Bad, Caspase‐9, nuclear factor‐κB, glycogen synthase kinase‐3, forkhead transcription factor Foxo1, p21Cip1, and p27Kip1, which collectively govern cell proliferation, differentiation, apoptosis, and migration [42, 43]. This pathway is tightly regulated by lipid phosphatases like Phosphatase and Tensin Homolog (PTEN), which dephosphorylates PIP3 to antagonize AKT activation [44]. However, impaired PTEN expression or function in CRC leads to enhanced pathway activity and accelerated tumor growth [45]. Notably, ARG2 has been reported to induce PI3K/AKT signaling in smooth muscle cells [46], and its interaction with this pathway is supported by studies in thyroid cancer and melanoma, where ARG2 ablation reduces AKT expression, promotes apoptosis, and decreases proliferation markers [14, 16]. Collectively, the PI3K/AKT pathway is a central signaling network in CRC, and its aberrant activation enhances cell growth, survival, and metabolism, conferring a proliferative advantage to cancer cells [47]. Our findings position ARG2 as a critical regulator of this pathway, driving CRC pathogenesis through enhanced PI3K/AKT signaling.

A notable limitation of our study lies in the clinical cohort characteristics, which restrict the generalizability of our findings to broader treatment contexts. Specifically, all included patients lacked pre‐surgery antitumor treatment (such as neoadjuvant chemotherapy or radiotherapy), precluding an analysis of how ARG2 expression might correlate with pretreatment status or respond to neoadjuvant interventions. Additionally, the sample size of our cohort limited our ability to perform stratified analyses on the impact of distinct postoperative adjuvant regimens (e.g., different chemotherapeutic combinations or targeted therapies) on the relationship between ARG2 expression and patient prognosis. These gaps highlight the need for future studies leveraging larger, multicenter cohorts that include both pretreated patients and those receiving diverse postoperative treatments. Such research would not only clarify ARG2's potential associations with treatment history but also validate its prognostic value across varied therapeutic contexts, enhancing its translational relevance for clinical decision‐making.

In conclusion, we demonstrated that ARG2 is highly expressed at both mRNA and protein levels in CRC and is associated with dMMR status. Functionally, ARG2 enhances CRC cell proliferation, migration, and invasion by inducing EMT and activating the PI3K/AKT pathway. Furthermore, ARG2 modulates the immune microenvironment and chemotherapeutic sensitivity.

## Author Contributions

**Yueyan Zhang:** data curation, investigation, validation, writing – original draft. **Ning Qu:** data curation, formal analysis, visualization, writing – original draft. **Jiale Mei:** investigation, methodology, validation. **Caixia Mo:** investigation, methodology, resources. **Luojuan Wei:** investigation, software, validation. **Haixian Shen:** investigation, software, visualization. **Shanfei Zhou:** investigation, resources, software. **Jinmin Hu:** validation, visualization, formal analysis. **Wenqi Luo:** conceptualization, funding acquisition, project administration, supervision, writing – review and editing. **Xianwei Mo:** conceptualization, funding acquisition, project administration, supervision, writing – review and editing.

## Funding

This work was supported by the Key Research and Development Program of Guangxi (AB23026079) and the College Students' Innovative Entrepreneurial Training Plan Program (S202410598217).

## Disclosure

The authors have nothing to report.

## Ethics Statement

Approval of the research protocol by an Institutional Review Board: Experiments involving patients were approved by the Ethics Committee of the Guangxi Medical University Cancer Hospital (KY2025663). All patients agreed to participate in this work.

## Conflicts of Interest

The authors declare no conflicts of interest.

## Supporting information


**Table S1:** Correlations between ARG2 expression in CRC tissues and the clinicopathologic characteristics of patients from our cohort.

## Data Availability

The article includes all data in the study, and further inquiries can be directed to the corresponding authors.
