# Supplementary material for: Arginase 2 Promotes Colorectal Cancer Metastasis via PI3K/AKT Pathway Activation and Regulates Tumor Immune Infiltration
Source: Cancer Med. 2026 Feb 3;15(2):e71567. doi: 10.1002/cam4.71567 (PMC12868387; doi:10.1002/cam4.71567)
Supplement: Supplementary file 1 — Table S1: Correlations between ARG2 expression in CRC tissues and the clinicopathologic characteristics of patients from our cohort. [file CAM4-15-e71567-s001.docx]

Supplementary table 1:

Correlations between ARG2 expression in CRC tissues and the clinicopathologic characteristics of patients from our cohort

| Characteristic |  | Expression of ARG2 | | χ^2^ | P-value |
| --- | --- | --- | --- | --- | --- |
|  |  | Low | High |  |  |
| Sex | Male | 66 (63.5%) | 38 (36.5%) | 0.011 | 0.918 |
|  | Female | 42 (62.7%) | 25 (37.3%) |  |  |
| Age | ≤60 | 54 (61.4%) | 34 (38.6%) | 0.251 | 0.616 |
|  | >60 | 54 (65.1%) | 29 (34.9%) |  |  |
| Size | ≤4cm | 30 (62.5%) | 18 (37.5%) | 0.012 | 0.911 |
|  | >4cm | 78 (63.4%) | 45 (36.6%) |  |  |
| Cancer embolus | No | 75 (61.0%) | 48 (39.0%) | 0.145 | 0.703 |
|  | Yes | 27 (64.3%) | 15 (35.7%) |  |  |
| Nerve invasion | No | 69 (64.5%) | 38 (35.5%) | 0.496 | 0.481 |
|  | Yes | 36 (59.0%) | 25 (41.0%) |  |  |
| T | T1-T2 | 28 (70.0%) | 12 (30.0%) | 1.050 | 0.305 |
|  | T3-T4 | 80 (61.1%) | 51 (38.9%) |  |  |
| N | N0 | 60 (65.2%) | 32 (34.8%) | 0.363 | 0.547 |
|  | N1-N2 | 48 (60.8%) | 31 (39.2%) |  |  |
| M | M0 | 98 (63.6%) | 56 (36.4%) | 0.152 | 0.696 |
|  | M1 | 10 (58.8%) | 7 (41.2%) |  |  |
| Stage | Stage I-II | 56 (64.4%) | 31 (35.6%) | 0.111 | 0.739 |
|  | Stage III-IV | 52 (61.9%) | 32 (38.1%) |  |  |
| Mismatch repair | dMMR | 22 (47.8%) | 24 (52.2%) | 6.342 | 0.012 |
|  | pMMR | 84 (68.9%) | 38 (31.1%) |  |  |
| Tumor budding | Poorly | 70 (68.0%) | 33 (32.0%) | 2.568 | 0.109 |
|  | Moderate-High | 38 (55.9%) | 30 (44.1%) |  |  |

**Abbreviations:** dMMR, deficient mismatch repair; pMMR, proficient mismatch repair.
